# Supplementary material for: Therapeutic Intervention for Various Hospital Setting Strains of Biofilm Forming Candida auris with Multiple Drug Resistance Mutations Using Nanomaterial Ag-Silicalite-1 Zeolite
Source: Pharmaceutics. 2022 Oct 21;14(10):2251. doi: 10.3390/pharmaceutics14102251 (PMC9611151; doi:10.3390/pharmaceutics14102251)
Supplement: Supplementary file 1 [file pharmaceutics-14-02251-s001.zip › pharmaceutics-1966346-supplementary.pdf]

Therapeutic intervention for various hospital setting strains of biofilm forming *Candida auris* with multiple drug resistance mutations using nanomaterial Ag-Silicalite-1 zeolite

Hanan A Aldossary [1], Suriya Rehman [2], B. Rabindran Jermy [3], Reem AlJindan [4], Afra Aldayel [5], Sayed AbdulAzeez [6], Sultan Akhtar [7], Firdos Alam Khan [8], J. Francis Borgio [2,6,*] and Ebtesam Abdullah Al-Suhaimi [9,*]

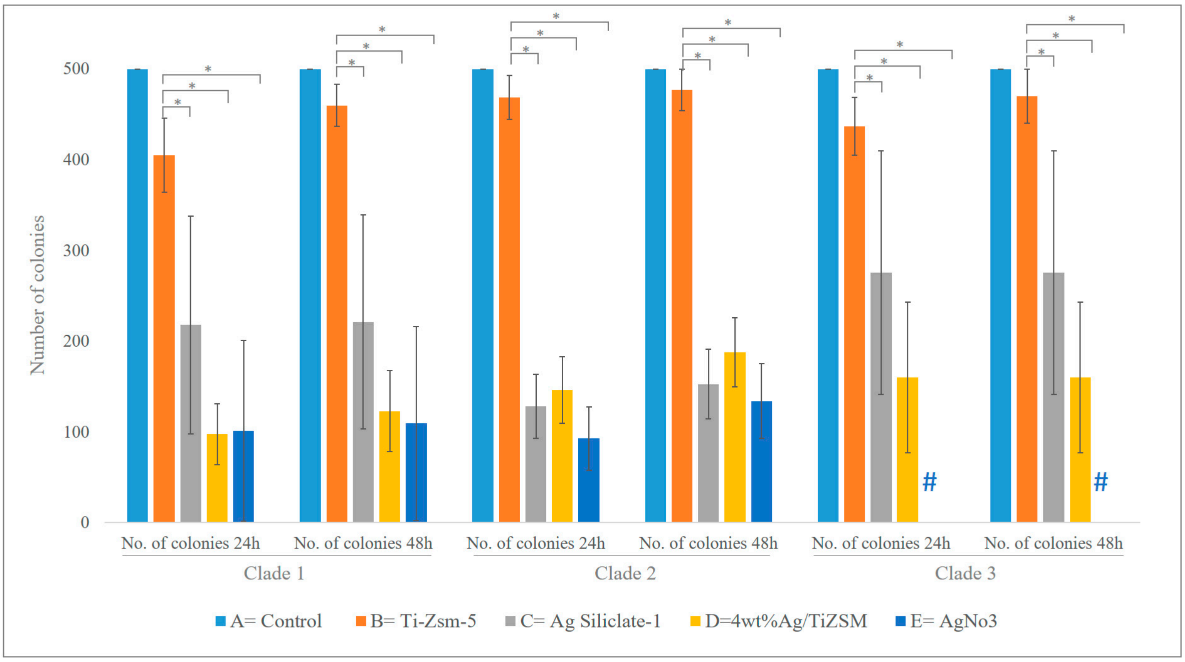

Supplementary Figure S1. Graph showing the effect of synthesized nanomaterial on planktonic cells of 3 different clades *C. auris* strains after 48 hours of incubation period. # Complete absence of growth. * $p < 0.01$.
